# Reporting Deaths Among Children Aged <5 Years After the Ebola Virus Disease Epidemic — Bombali District, Sierra Leone, 2015–2016

**DOI:** 10.15585/mmwr.mm6641a5

**Published:** 2017-10-20

**Authors:** Amanda L. Wilkinson, Reinhard Kaiser, Mohamed F. Jalloh, Mamudi Kamara, Dianna M. Blau, Pratima L. Raghunathan, Alpha Kamara, Umaru Kamara, Nathaniel Houston-Suluku, Kevin Clarke, Amara Jambai, John T. Redd, Sara Hersey, Brima Osaio-Kamara

**Affiliations:** ^1^Epidemic Intelligence Service, CDC; ^2^Child Health and Mortality Prevention Surveillance Network, Emory Global Health Institute, Emory University, Atlanta, Georgia; Center for Global Health, CDC; ^3^Division of Global Health Protection, Center for Global Health, CDC; ^4^Ministry of Health and Sanitation, Sierra Leone; ^5^eHealth Africa, Sierra Leone; ^6^CDC Country Office, Sierra Leone.

Mortality surveillance and vital registration are limited in Sierra Leone, a country with one of the highest mortality rates among children aged <5 years worldwide, approximately 120 deaths per 1,000 live births (*1**,**2*). To inform efforts to strengthen surveillance, stillbirths and deaths in children aged <5 years from multiple surveillance streams in Bombali Sebora chiefdom were retrospectively reviewed. In total, during January 2015–November 2016, 930 deaths in children aged <5 years were identified, representing 73.3% of the 1,269 deaths that were expected based on modeled estimates. The “117” telephone alert system established during the Ebola virus disease (Ebola) epidemic captured 683 (73.4%) of all reported deaths in children aged <5 years, and was the predominant reporting source for stillbirths (n = 172). In the absence of complete vital events registration, 117 call alerts markedly improved the completeness of reporting of stillbirths and deaths in children aged <5 years.

The 2016 National Civil Registration Act established a new authority in Sierra Leone responsible for recording vital events.* The act is an essential step toward improving national death reporting and registration, which are currently largely paper-based and limited in coverage. Improving death reporting is needed to enhance disease reporting, facilitate more rapid disease control, and enhance global health security. In March 2017, Sierra Leone implemented an electronic reporting system, Integrated Disease Surveillance and Response, which includes facility-based maternal mortality reporting (*3*). Discussions are ongoing regarding adding deaths among children aged <5 years to the reporting. Until further improvements in reporting systems are introduced, decision-makers must rely on modeled national mortality rate estimates for children aged <5 years.

Preparations are underway to implement a Child Health and Mortality Prevention Surveillance (CHAMPS)^†^ site in Bombali Sebora chiefdom, Bombali District, northern Sierra Leone (population approximately 161,000). CHAMPS seeks to generate high-quality cause-of-death data for children aged <5 years through multifaceted postmortem investigations. A baseline assessment of surveillance for stillbirths and deaths among children aged <5 years was conducted during 2015–2016 to guide surveillance strengthening and CHAMPS cause-of-death investigations.

The main objectives of the assessment were to assess the relative contributions of different reporting mechanisms to death ascertainment and to evaluate reporting completeness by comparing the number of documented deaths with national mortality estimates calculated from modeling. Eligible cases were defined as stillbirths and deaths among resident Bombali Sebora children aged <5 years that occurred from January 1, 2015 through November 25, 2016.

Data from three existing reporting streams were used in this analysis. The first was the 117 telephone alert system; the second included records from eight Bombali Sebora health facilities, and the third contained vital records from the Makeni Office of Births and Deaths. The toll-free 117 phone alert system was established in August 2014 to allow rapid notification and investigation of suspected Ebola cases and all deaths from the community (*4*). After the epidemic, the phone alert system remained in place under a policy of mandatory death reporting and Ebola testing, and changed to report all deaths in July 2016. Telephone alert calls peaked in October 2014 at >11,000 per week; by December 2016, calls had decreased to <500 per week.

Data on stillbirths and deaths in children aged <5 years from handwritten health facility and Office of Births and Deaths records were abstracted into prepared excel spreadsheets. Deaths were linked across data sources using the child’s name (or the mother’s name for stillbirths), date of death or stillbirth, age, sex, residence address, and location of death as identifiers. Because denominators for calculating rates and expected numbers of stillbirths and deaths among children aged <5 years in Bombali Sebora were unavailable, expected numbers of stillbirths and deaths were calculated^§^ using national mortality rates for all children aged <5 years (approximately 120 of 1,000 live births)(*1**,**2*), infants (death during the first year of life; 87 per 1,000), and neonates (deaths during the first 28 days of life; 35 per 1,000 live births), national stillbirth rate (27 per 1,000 births)(*4*), and crude estimates of the birth rate (34.2 births per 1,000 population) (*5*,*6*).

After consolidation and deduplication of records identified in the three data sources, 172 unique stillbirths and 930 unique deaths among children aged <5 years were identified, including 249 neonatal deaths (27%), 247 (27%) deaths in infants aged 1–11 months, and 434 (47%) deaths in children aged 1–4 years (Figure) (Table). Death reports from health facilities and vital records were lowest in early 2015, when multiple facilities remained closed because of the Ebola epidemic. There was minimal overlap among the different reporting streams: only 11% of deaths were reported through more than one source (Figure). The majority of deaths (600; 65%) were documented through 117 phone alert only, followed by 20% (187) through health facilities only, and 5% (45) through vital records only. The proportion of deaths reported by phone alert decreased from 81% in 2015 to 65% in 2016. The percentage of expected deaths that were reported declined from 92% in 2015 to 53% in 2016. The number of infant deaths reported varied most from the number expected: reported infant deaths were 70% of expected in 2015 and 37% of expected in 2016.

**FIGURE:**
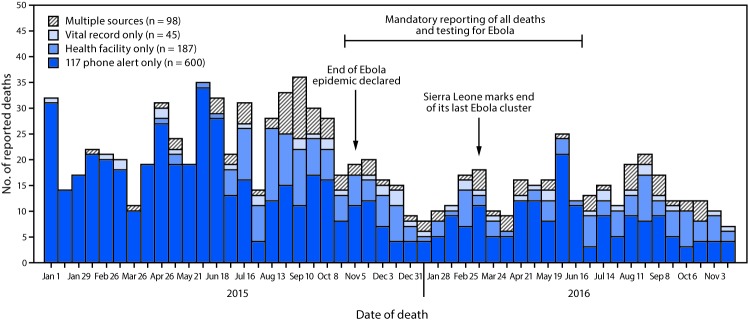
Number of reported deaths in children aged <5 years (N = 930), by reporting source* — Bombali Sebora chiefdom, Bombali District, Sierra Leone, January 2015–November 2016 **Abbreviation**: Ebola = Ebola virus disease. * Reported deaths among children aged <5 years were ascertained through one or more of the following reporting streams: 1) the 117 phone alert system established during the Ebola epidemic; 2) records from eight Bombali Sebora health facilities, and 3) vital records from the Makeni Office of Births and Deaths.

**TABLE:** Number of expected and reported stillbirths and deaths among children aged <5 years, by year — Bombali Sebora chiefdom, Bombali District, Sierra Leone, January 2015–November 2016

Reported and expected deaths	Stillbirths	Total deaths among children aged <5 years	Infant deaths (0–12 months)	Neonatal deaths (0–27 days)
**Jan–Dec 2015**				
Reported*	91	**606**	334	161
Expected^†^	145	**662**	480	193
Ratio (reported/expected)	0.63	**0.92**	0.70	0.83
**Jan**–**Nov 2016**				
Reported*	81	**324**	162	88
Expected^†^	132	**607**	440	177
Ratio (reported/expected)	0.61	**0.53**	0.37	0.50
**Overall**				
Reported*	172	**930**	496	249
Expected^†^	277	**1,269**	920	370
Ratio (reported/expected)	0.62	**0.73**	0.54	0.67

* Reported stillbirths and deaths among children aged <5 years were ascertained through one or more of the following reporting streams: 1) the 117 phone alert system established during the Ebola virus disease epidemic; 2) records from eight Bombali Sebora health facilities, and 3) vital records from the Makeni Office of Births and Deaths.

^†^ Expected numbers were estimated using national under-5, infant, and neonatal mortality rates (120, 87, and 35 deaths per 1,000 live births, respectively), stillbirth rate (SBR) (27 stillbirths per 1,000 births), and crude birth rate (34.2 births per 1,000 population). Deaths in children aged <5 years were calculated using the formula: deaths = live births x mortality rate; stillbirths were calculated using the formula: stillbirths = live births x SBR /(1 – SBR).

Among an expected 277 stillbirths, 172 (62%) were reported. A majority of stillbirths were reported through phone alert (107; 62%) or health facility records only (53; 31%), with one stillbirth identified through a death record, and 11 (6%) by more than one source. Stillbirth reporting patterns were inconsistent over time and information on gestational age at delivery was rarely available.

## Discussion

On the basis of the national estimates used for these analyses, stillbirths and deaths in children aged <5 years are underreported in Bombali Sebora chiefdom; use of all available sources is needed to improve death reporting in this chiefdom. The 117 phone alert system captured community-based deaths and stillbirths not recorded through health facility and vital records.

The findings in this report are subject to at least three limitations. First, expected stillbirths and deaths were calculated based on point estimates; therefore, comparisons with reported cases should be interpreted with caution. Second, case misclassification might have occurred because of age estimation errors and inconsistent application of a standard case definition for stillbirths. Stillbirth classification was largely self-defined by reporters, and there is a high likelihood that certain spontaneous miscarriages and early neonatal deaths were included. Finally, when interpreting trends in stillbirth and child mortality over time, it is important to note that separating the effects of the Ebola epidemic on child mortality from changes in reporting and documentation was not possible. For example, the July 2016 change to nonmandatory 117 death reporting would be expected to lead to a decline in death reporting through this source; however, the contribution of this change to the proportion of expected deaths that were reported could not be determined. Despite these limitations, the findings in this report indicate that surveillance in this setting can be strengthened by using multiple data sources, which together capture both community and facility deaths. These findings also demonstrate that community-based reporting strategies, such as phone alerts, can be implemented in countries with incomplete death registration to supplement vital events records. 

Sierra Leone continues to use the 117 phone alert system to improve national disease surveillance and plans are in place to include the system for death reporting as part of enhanced child mortality surveillance through CHAMPS in Sierra Leone. Additional strategies are needed to improve overall child morality surveillance and to promote post-Ebola community participation in death reporting using the 117 phone alert system.

**Summary**

**What is already known about this topic?**

Inadequate vital events registration is common in low- and middle-income countries, including Sierra Leone. To estimate child mortality in the absence of reliable vital records, additional data sources are needed.

**What is added by this report?**

Assessing multiple death reporting streams, including the 117 phone alert system established during the Ebola virus disease outbreak, improved ascertainment of deaths among children aged <5 years and stillbirths.

**What are the implications for public health practice?**

Community-based reporting strategies (e.g. phone alerts) can be implemented in countries with incomplete death registration to supplement vital events records and strengthen child mortality surveillance.

## Footnotes

* http://www.parliament.gov.sl/dnn5/LinkClick.aspx?fileticket=hUAMYdkuwpU%3D&tabid=79&mid=650.

† https://champshealth.org/.

§ Expected deaths in children aged <5 years were calculated using the following formula: deaths = live births x national published mortality rate for children aged <5 years and stillbirth rate (SBR). Expected stillbirths were calculated using the following formula: stillbirths = live births x stillbirth rate (SBR)/(1 – SBR).
